# Supplementary material for: Immobilization of Cadmium by Molecular Sieve and Wollastonite Is Soil pH and Organic Matter Dependent
Source: Int J Environ Res Public Health. 2021 May 12;18(10):5128. doi: 10.3390/ijerph18105128 (PMC8150881; doi:10.3390/ijerph18105128)
Supplement: Supplementary file 1 [file ijerph-18-05128-s001.zip › ijerph-1199605-Supplementary.pdf]

SUPPLEMENTARY

Immobilization of cadmium by molecular sieve and wollastonite is soil pH and organic matter dependent

Meiliang Dong [1,2], Rong Huang [1,2], Peng Mao [1], Long Lei [1,2], Yongxing Li [1], Yingwen Li [1], Hanping Xia [1]
Zhian Li [1,3,*], Ping Zhuang [1,3,*]

[1] Key Laboratory of Vegetation Restoration and Management of Degraded Ecosystems, South China Botanical Garden, Chinese Academy of Sciences, Guangzhou 510650, China

[2] University of Chinese Academy of Sciences, Beijing 100049, China

[3] Southern Marine Science and Engineering Guangdong Laboratory (Guangzhou), Guangzhou 511458, China

Table S1 Characteristics of two amendments.

Characteristics	4A molecular sieve	Wollastonite
Ingredients	36.98% SiO_2, 28.84% Al_2O_3, 15.84% Na_2O	45.18% SiO_2, 42.76% CaO, 3.04% MgO
pH	11.87	8.43
Diameter (um)	19	14
Bore diameter (nm)	0.4	—
Surface area (m^2 g^{-1})	600-800	—
Manufacturer	Shanghai Molsion Molecular Sieve Co., Ltd.	Jiangxi Shengtai Chemical Co., Ltd.

Table S2 Tessier sequential extraction method of heavy metals in soil

	Reagent and extraction conditions
Exchangeable fraction (F1)	8 mL of 1 M $MgCl_2 \cdot 6H_2O$ (pH 7.0), 1 h continuous shaking under 25 °C
Bound to carbonates/acid soluble fraction (F2)	8 mL of 1 M CH_3COONa (adjusted to pH 5 with CH_3COOH), 5 h continuous shaking under 25 °C
Bound to iron and manganese oxides/reducible fraction (F3)	20 mL of 0.04 M $NH_2OH \cdot HCl$ in 25% CH_3COOH, 96°C in a water bath for 4 h
Bound to organic matter/oxidizable fraction (F4)	3 mL 0.02 M HNO_3 + 5 mL 30 % H_2O_2 (adjusted to pH 2 with HNO_3), 85°C in a water bath for 2 h, shake for 1 minute every half hour; add 3 mL 30 % H_2O_2 (adjusted to pH 2 with HNO_3), 85°C-water bath for 3 h, shake for 1 minute every half hour; after cooling, add 5ml 3.2 M CH_3COONH_4, shake for 30 minutes
Residual fraction (F5)	1ml $HClO_4$ +5ml HF, digest using microwave oven

Note: Shaking was applied at 200 rpm; Extraction of the residual fraction in the microwave was equal to the pseudo-total extraction method.

Table S3 Effects of adding 440 mg kg^{-1} Si on several elements concentrations of amaranth shoot in six soils.

Soils	Treatments	K g/kg	Mg g/kg	Cu mg/kg	Zn mg/kg	Mn mg/kg	Pb mg/kg	Ca g/kg	Na g/kg
Soil 1	Control	37.7±2.6ab	8.1±0.4a	16.4±1.5a	530±91.3a	37.1±3.6a	8.0±1.5ab	35.1±3.2ab	2.1±0.4b
	MS	31.5±5b	7.3±0.6a	13.9±2.3a	320±15.3b	34.1±4.6ab	10.3±1.2a	31.3±0.8b	12±0.4a
	WS	43.6±1.2a	8.6±0.7a	16.1±1.9a	218±22.0b	25.7±2.0b	6.5±0.4b	38.4±0.5a	2.5±0.3b
Soil 2	Control	27.4±3.1a	3.0±0.4a	14.4±1.8a	1744±78.1a	345±29.7a	14.8±0.8a	19.4±1.2b	2.7±0.4b
	MS	27.4±2.8a	2.9±0.2a	10.0±2.0a	1060±173b	164±14.3b	13.6±2.2a	20.1±1.2b	14.8±2.7a
	WS	31.4±2.5a	3.5±0.5a	14.6±2.5a	1108±68.1b	127±4.9b	9.4±0.4b	29.5±3.2a	2.1±0.2b
Soil 3	Control	37.3±3.2a	7.5±0.3b	20.4±0.9a	402±23.8a	101±2.0a	3.7±0.7a	27.7±2.4ab	2.7±0.05b
	MS	42.4±2.2a	7.1±0.9b	20.6±2.6a	267±19.3b	97.8±7.4a	3.8±0.2a	24.8±2.2b	6.1±1.0a
	WS	36.5±3.8a	9.6±0.6a	18.7±1.1a	306±21.5b	74.2±11.4b	2.2±0.3b	32.9±4.6a	1.7±0.1b
Soil 4	Control	25.2±2.9a	6.8±0.6a	17.0±3.6a	198±30.2a	77.8±8.1a	7.9±1.2a	27.6±3.1a	4.2±0.7b
	MS	26.6±2.6a	4.3±0.6b	10.8±1.3b	89.3±9.6b	31.4±5.5b	4.2±0.6b	18.5±2b	8.8±1.1a
	WS	30.7±2.9a	4.6±0.5b	12.8±1.2ab	70.1±10.3b	19.5±1.5b	6.8±1.3ab	26.1±2.8a	1.6±0.03c
Soil 5	Control	32.7±6.6a	5.5±0.4a	13.7±1.4a	203±34.6a	1476±161a	5.3±0.6a	28.5±2b	2.3±0.1b
	MS	41.6±2.8a	4.8±0.4a	14.0±0.5a	70.9±9.6b	173±3.0b	2.8±0.3b	25.5±1.8b	17.6±2.3a
	WS	42.5±2.0a	5.4±0.8a	15.7±2.3a	71.1±5.7b	72.2±13.2b	5.4±0.2a	37.4±3.6a	1.7±0.03b
Soil 6	Control	39.1±6.0a	18.3±1.3a	19.6±2.7a	380±47.7a	73.4±10.8a	24.2±2.5a	29.9±1.6a	1.3±0.1b
	MS	36.8±4.7a	20.1±0.5a	19.9±3.8a	336±56.5a	76.8±14.2a	17.5±2.8a	28.2±0.9a	3.6±0.5a
	WS	38.2±4.4a	17.4±2.4a	17.3±0.9a	341±33.4a	63.7±3.1a	23.5±3.4a	29.8±1.8a	2.1±0.3b

Control, non-amendments control; MS, 4A molecular sieve; and WS, wollastonite. Data are means ± SE (n=4). Same letters beside the means refer to no significant difference according to Tukey honestly significant difference test ($p < 0.05$).

Table S4 Effects of adding 440 mg kg^{-1} Si on several elements concentrations of amaranth root in six soils.

Soils	Treatments	K g/kg	Mg g/kg	Cu mg/kg	Zn mg/kg	Mn mg/kg	Pb mg/kg	Ca g/kg	Na g/kg
Soil 1	Control	31.7±3.8a	4.6±0.8a	19.7±3.0a	561.3±101.3a	30.7±3.7a	26.6±4.5a	17.6±1.9ab	3.2±0.4b
	MS	30.7±1.6a	4.0±0.7a	22.2±1.0a	310.3±64.8b	25.0±1.7ab	22.7±4.1ab	15.5±1.4b	20.1±1.2a
	WS	36.4±7.1a	4.8±0.6a	25.7±4.1a	237.6±23.4b	18.0±0.6b	17.0±2.6b	22.5±1a	3.0±0.4b
Soil 2	Control	28.1±3.9a	1.1±0.2a	18.8±1.0ab	2573.7±326.0a	109.1±15.8a	125.1±20.0a	12.7±0.2b	4.0±0.6b
	MS	13.4±1.8b	0.7±0.1b	13.5±1.4b	1592.3±171.6b	69.3±8.7b	72.9±10.5b	10.2±0.5b	23.8±1.7a
	WS	23.4±2.0a	1.3±0.06a	22.5±3.4a	1621.3±272.7b	37.2±5.5c	60.1±11.2b	17.4±1.4a	3.4±0.2b
Soil 3	Control	31.9±1.9a	3.9±0.1a	33.8±4.5a	414.3±15.0a	63.4±2.8a	34.4±3.5a	16.2±2.3a	3.2±0.4b
	MS	24.9±1.9b	3.9±0.5a	33.6±6.5a	263.8±18.6b	56.3±5.1a	32.2±5.3ab	13±2.0a	17.5±0.5a
	WS	28.7±3.1ab	4.4±0.5a	39.6±4.3a	267.3±43.5b	42.2±5.5b	25.2±4.4b	16.9±2.6a	3.1±0.5b
Soil 4	Control	29.4±2.2a	5.5±0.4a	23.4±4.1a	191.2±7.7a	60.3±12.1a	7.3±0.9a	15.4±12.4b	3.1±0.3b
	MS	21.5±1.6b	5.1±0.7a	20.6±2.6a	86.9±9.5b	34.2±5.9b	6.5±0.2a	13.7±2.3b	13.4±1.7a
	WS	32.6±4.4a	5.3±0.7a	22.7±1.2a	80.5±9.1b	19.8±1.1b	6.4±0.9a	19.6±1.4a	2.6±0.2b
Soil 5	Control	30.7±1.1a	3.5±0.5a	85.6±10.8a	213.5±36.9a	622.1±54.3a	14.5±1.0a	18.5±1.2b	4.5±0.2b
	MS	22.3±0.3b	4.4±0.8a	51.6±2.8b	97.4±2.0b	150.6±18.5b	10.3±1.5b	18.5±2.3b	19±2.5a
	WS	31.8±5.6a	4.4±0.6a	36.0±4.2b	75.5±6.2b	59.5±8.7c	8.0±0.8c	29.5±3.3a	4.0±0.6b
Soil 6	Control	36.2±4.7a	11.1±0.7a	37.4±8.2a	772.0±106.5a	84.6±13.7a	109.0±16.5a	16.4±2.6a	2.2±0.2b
	MS	23.8±1.7b	9.8±0.5a	32.9±7.9a	546.1±77.8b	77.3±7.3a	91.0±9.9a	16.2±1.3a	16.7±1.3a
	WS	35.5±2.8a	9.8±1.5a	33.7±2.4a	668.0±58.4ab	69.3±8.8a	92.0±6.6a	19.3±3.0a	3.9±0.2b

Control, non-amendments control; MS, 4A molecular sieve; and WS, wollastonite. Data are means ± SE (n=4). Same letters beside the means refer to no significant difference according to Tukey honestly significant difference test ($p < 0.05$).

Table S5 Effects of different added dosages of amendments on several elements concentrations of amaranth shoot in soil 3.

Treatments	Si mg/kg	K g/kg	Mg g/kg	Cu mg/kg	Zn mg/kg	Mn mg/kg	Pb mg/kg	Ca g/kg	Na g/kg
Control	0	37.3±3.2ab	7.5±0.3b	20.4±0.9a	402±23.8a	101±2.0a	3.7±0.7bc	27.7±2.4ab	2.7±0.05d
MS	220	39.5±2.8ab	8.1±1.4ab	18.6±1.4ab	342±25.1bc	102±1.7a	3.7±0.3bc	25.1±1.7ab	3.6±0.3cd
	440	42.4±2.2a	7.1±0.9bc	20.6±2.6a	267±19.3d	97.8±7.4a	3.8±0.2b	24.8±2.2ab	6.1±1.0c
	660	33.3±3.6b	5.4±0.4cd	15.8±2.2b	177±18.5ef	72.5±13.0bc	5.7±0.4a	19.2±3.1b	8.7±1.5b
	880	33.4±1.3b	4.0±0.7d	14.7±1.2b	130±10.0f	54.5±9.0cd	4.5±0.4ab	20.7±1.6b	12.3±1.7a
WS	220	39.4±2.9ab	8.4±0.3ab	18±1.4ab	370±34.3ab	87.6±9.7ab	3.9±0.9b	30.7±2.2ab	2.2±0.2d
	440	36.5±3.8ab	9.6±0.6a	18.7±1.1ab	306±21.5cd	74.2±11.4bc	2.2±0.3c	32.9±4.6a	1.7±0.1d
	660	36.9±2.3ab	8.8±0.4ab	18.3±1.1ab	199±27.1e	49.3±5.5de	2.2±0.3c	34.5±0.6a	1.4±0.2d
	880	40.3±5.5ab	8.3±0.7ab	18.6±2.3ab	121±18.7f	30.5±1.1e	4.4±0.6ab	35.5±2.4a	1.6±0.2d

Control, non-amendments control; MS, 4A molecular sieve; and WS, wollastonite. Data are means ± SE (n=4). Same letters beside the means refer to no significant difference according to Tukey honestly significant difference test ($p < 0.05$).

Table S6 Effects of different added dosages of amendments on several elements concentrations of amaranth root in soil 3.

Treatments	Si<br>mg /kg	K<br>g/kg	Mg<br>g/kg	Cu<br>mg/kg	Zn<br>mg/kg	Mn<br>mg/kg	Pb<br>mg/kg	Ca<br>g/kg	Na<br>g/kg
Control	0	31.9±1.9abc	3.9±0.1ab	33.8±4.5ab	414.3±15.0a	63.4±2.8a	34.4±3.5ab	16.2±2.3abc	3.2±0.4c
MS	220	25.5±3.7de	3.7±0.5b	28.5±2.7ab	374.0±54.0a	59.0±4.4a	35.3±0.6a	12.6±0.9c	12±0.9b
	440	24.9±1.9de	3.9±0.5b	33.6±6.5ab	263.8±18.6b	56.3±5.1ab	32.2±5.3ab	13±2.0c	17.5±0.5a
	660	20.5±2.9e	3.6±0.3b	28.7±3.9ab	221.6±25.0bc	54.0±6.0ab	25.1±3.2bcd	11.4±1.4c	18.6±2.7a
	880	23.4±2.4de	5.3±0.8a	38.2±4.7a	127.3±22.2c	36.1±6.8c	25.6±4.9bc	12.8±1.8c	21.6±3.0a
WS	220	31.8±0.55abc	4.0±0.6ab	39.6±3.9a	406.8±70.4a	60.7±5.6a	28.3±3.8abc	15.6±1.2bc	3.2±0.3c
	440	28.7±3.1bcd	4.4±0.5ab	39.6±4.3a	267.3±43.5b	42.2±5.5bc	25.2±4.4bc	16.9±2abc	3.1±0.5c
	660	35.0±2.5ab	4.8±0.5ab	25.7±2.8b	221.2±12.2bc	30.1±0.8cd	20.3±3.8cd	20.8±3ab	2.6±0.05c
	880	36.3±4.7a	4.9±0.8ab	24.3±0.2b	176.2±38.5bc	17.5±2.1d	15.8±2.5d	22.1±2.2a	2.1±0.3c

Control, non-amendments control; MS, 4A molecular sieve; and WS, wollastonite. Data are means ± SE (n=4). Same letters beside the means refer to no significant difference according to Tukey honestly significant difference test ($p < 0.05$).

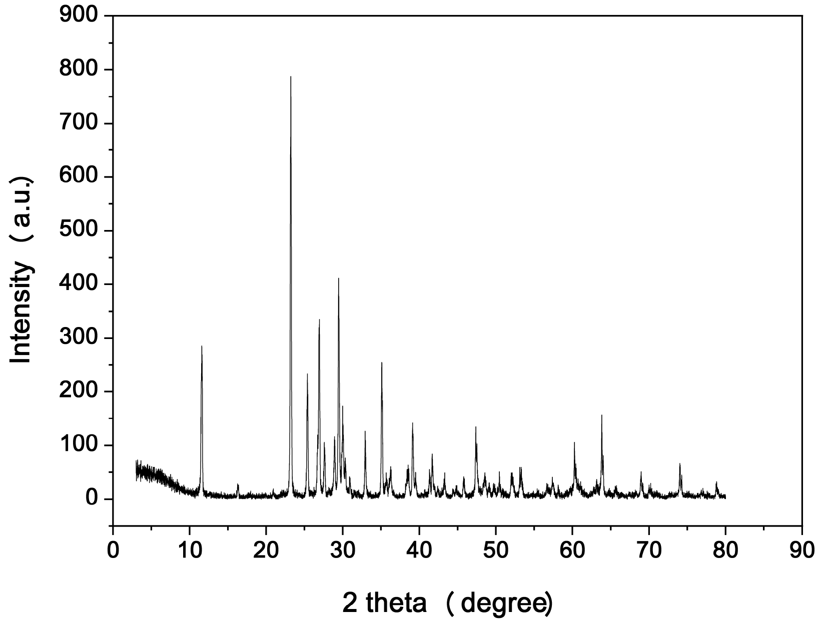

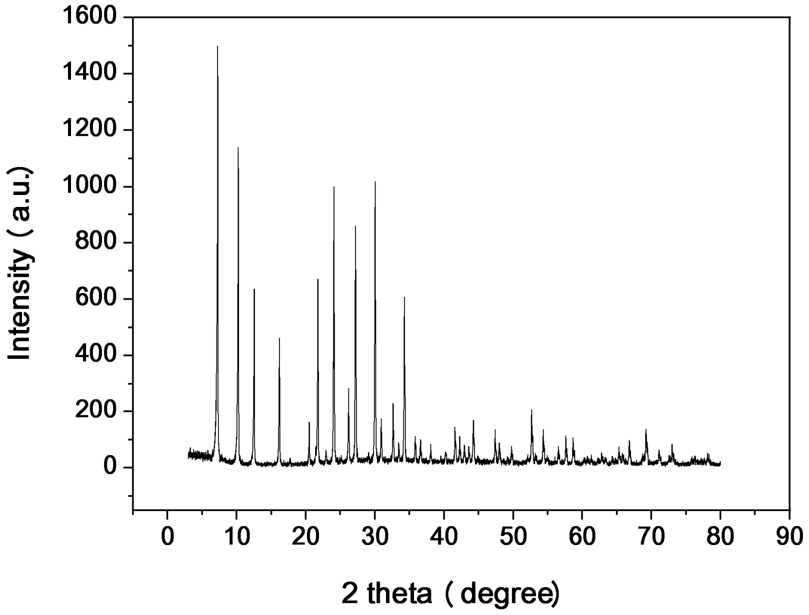

Fig. S1 The XRD reflections of wollastonite materials were indexed to 4Å-molecular sieve（PDF No. 39-0223）and Wollastonite-2M (JCPDS No.43460), which indicates the high purity of sample and absence of any mineral impurities detectable by XRD.

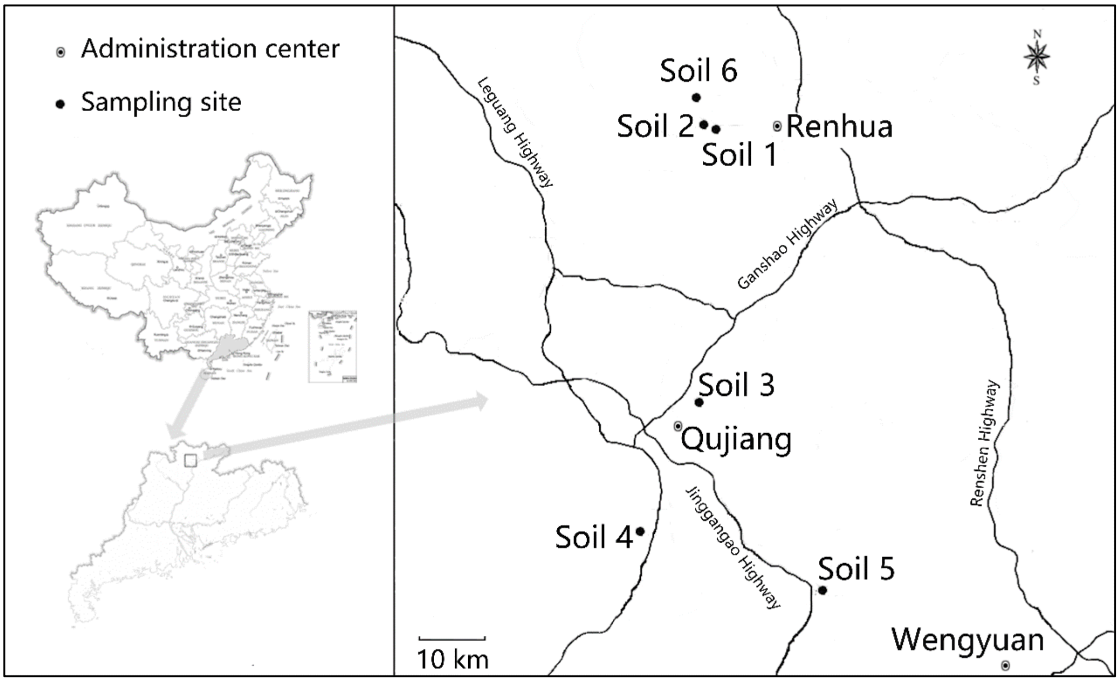

Fig. S2 Location of the sampling sites.

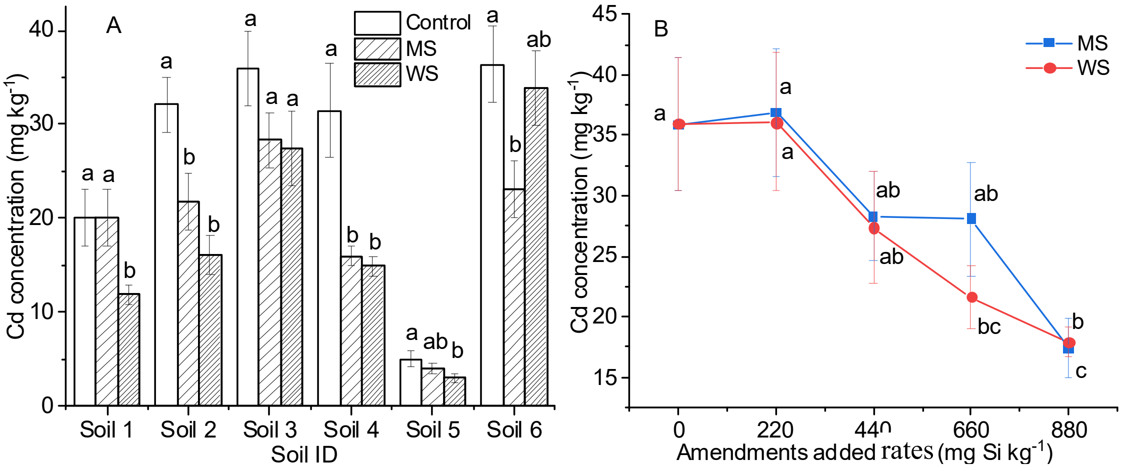

Fig. S3 Cd concentration of amaranth root in six Cd-contaminated soils under 440 mg kg^{-1} Si treatments (A) and in soil 3 under 0, 220, 440, 660 and 880 mg kg^{-1} Si added dosages (B). Control, non-amendments treatment; MS, 4A molecular sieve; WS, wollastonite. Data are means ± SE (n=4). Different letters above the adjacent bars or lines denote a significant ($p < 0.05$) difference among the treatments in the same soil.

Batch sorption experiments

Batch adsorption experiments were set-up to evaluate the effect of increasing Ca^{2+} concentration on the Cd adsorption capacity of amended and non-amended soils. Stock solutions (1000 mg L^{-1}) of Cd and Ca were prepared from $Cd(NO_3)_2$ and $Ca(NO_3)_2$, respectively. Binary solutions were prepared from the stock solutions with Ca: Cd initial molar concentration ratios of 10: 1, 20: 1, 30: 1, 40: 1, 50: 1, and 60: 1, as well as an initial Cd concentration of 5 mg L^{-1}. The pH of all the binary solutions was measured, but not adjusted, at a value of 5.3 ± 0.1. Mixtures containing 50 ml of binary solution and 1 g of soil (with 220 mg kg^{-1} of either MS- or WS-amended soil 3, as well as unamended soil 3) were shaken for 16 h at 25 ± 2 °C in quadruplicates. The mixtures were then centrifuged for 10 min (4000 r/min, 25 °C) and filtered. Supernatants were diluted for metal concentration analysis by atomic absorption spectroscopy (AAS, contrAA 800, Analytik Jena, Germany). The Cd adsorption capacity (Qe) of soil was presented as follows:

$$\mathrm{Qe} = \frac{[(Co-Ce)\mathrm{V}]}{\mathrm{m}}$$

where *Co* was the initial concentration, *Ce* was the equilibrium metal concentrations, m (g) was the mass of the applied soils, and V (mL) was the volume of the metal solution. Results are shown in Fig. S5.

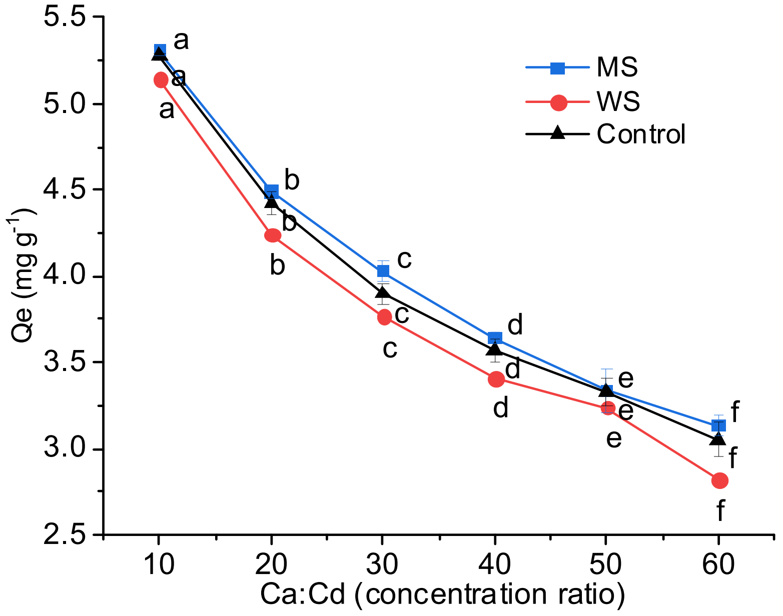

Fig. S4 Cd adsorption capacity of amended and unamended soil 3 in binary solutions with Ca: Cd molar concentration ratios of 10: 1, 20: 1, 30: 1, 40: 1, 50: 1, 60: 1. Control, non-amendments treatment; MS, 220 mg kg^{-1} Si (4A molecular sieve) amended soil 3; WS, 220 mg kg^{-1} Si (wollastonite) amended soil 3. Data are means ± SE (n=4). Different letters above the adjacent bars or lines denote a significant ($p < 0.05$) difference among the treatments in the same soil.
